# Use of Fourier-Domain Optical Coherence Tomography to Evaluate Anterior Stromal Opacities in Donor Corneas

**DOI:** 10.1155/2013/397680

**Published:** 2013-03-28

**Authors:** Matthew R. Bald, Christopher Stoeger, Joshua Galloway, Maolong Tang, Jeffrey Holiman, David Huang

**Affiliations:** ^1^Center for Ophthalmic Optics & Lasers, Casey Eye Institute and Department of Ophthalmology, Oregon Health & Science University, 3375 SW Terwilliger Boulevard, Portland, OR 97239-4197, USA; ^2^Lions VisionGift, Portland, OR 971214-5303, USA

## Abstract

*Purpose*. To evaluate Fourier-domain optical coherence tomography (FD-OCT) as an adjunct to traditional slit lamp examination of donor corneas with suspected Anterior Stromal Opacities. *Methods*. Seven corneas suspected of having anterior stromal opacities by slit lamp examination were evaluated with FD-OCT. Each cornea was evaluated to confirm the presence of opacity and, if present, the depth of opacity was measured. *Results*. The opacity depth ranged from 82 **μ**m to 624 **μ**m. The initial slit lamp impressions of five of the seven corneas were confirmed by OCT. In two corneas, the OCT findings were different from the initial slit lamp impressions. Slit lamp examination of the first cornea gave the impression of anterior stromal scarring, but OCT showed that the opacity was limited to the epithelium. Slit lamp examination of the second cornea suggested opacity limited to the epithelium, but OCT identified significant sub-Bowman's scarring. In all cases, the Eye Bank Technicians reported that the location and depth of corneal opacity were more sharply defined by OCT than by slit lamp. *Conclusion*. The high resolution of OCT makes it easier to determine the location of corneal opacities compared to slit lamp examinations. This enhanced visualization can improve decisions regarding transplant suitability of donor corneas.

## 1. Introduction

Eye banks currently employ a number of methods for assessing donor corneal tissue, including penlight exam, specular microscopy, slit lamp biomicroscopy, medical record review, and family interview [1]. Although these evaluation techniques are largely successful in identifying the contraindications that bar corneas from use in procedures such as penetrating keratoplasty (PK), anterior lamellar keratoplasty (ALK), and endothelial keratoplasty (EK), some eye banks have recently begun adopting Fourier-domain optical coherence tomography (FD-OCT) as a way of supplementing their standard procedures for tissue appraisal. 

Providing high-resolution, cross-sectional images of internal biological microstructures, OCT was first used by ophthalmologists to image the retina [2–4] and then more recently the anterior segment [5–7]. It has also been used to investigate the results of refractive surgeries in situ [8–10] and screen donor corneas for previous refractive surgeries such as laser in situ keratomileusis (LASIK) and photorefractive keratectomy (PRK) [11–13]. In addition, OCT has proven to be a useful tool in evaluating different eye bank tissue processing techniques for lamellar keratoplasty [14] and optimizing the thickness of corneal grafts prepared by microkeratome [15].

OCT's unique ability to accurately map corneal thickness over a wide area and provide precise measurements of stromal opacities while avoiding tissue contamination [16, 17] makes it a potentially valuable instrument for screening donor corneas. This potential is bolstered by the Eye Bank Association of America's (EBAA) 2005 decision to expand the criteria for acceptable tissue: [1] corneas that had been deemed unsuitable for PK because of anterior scarring, central pterygia, or corneal refractive surgeries such as Radial Keratotomy (RK) are now being effectively used in EK procedures [18, 19]. It is therefore increasingly important that each donor cornea is evaluated both accurately and efficiently in order to make the best use of all available tissue. The use of FD-OCT as an adjunct to standard tissue evaluation methods may provide eye banks with the information necessary to improve their decision making processes and ensure that no donor cornea is wasted or misused. This study examined the utility of FD-OCT as a supplemental tool in evaluating donor corneas suspected of having anterior stromal opacities.

## 2. Materials and Methods

### 2.1. Standard Tissue Evaluation

Lions VisionGift (formerly the Lions Eye Bank of Oregon) identified seven corneas as having potential anterior stromal pathology. The corneoscleral rims were immersed in Optisol-GS (Bausch & Lomb, Irvine, CA, USA) inside a corneal viewing chamber (Krolman, Boston, MA, USA) throughout the evaluation process. Each tissue evaluation began with an endothelial cell count using the Konan EKA-04 Eye Bank Specular Microscope (Konan Medical, Inc, Hyogo, Japan) and was followed by slit lamp examination with the Haag Streit BX 900 (Haag Streit, Koeniz, Switzerland). The slit lamp examinations were observed by a minimum of two EBAA Certified Eye Bank Technicians.

### 2.2. OCT Scanning of Corneas with Anterior Stromal Pathology

**Figure 1:**
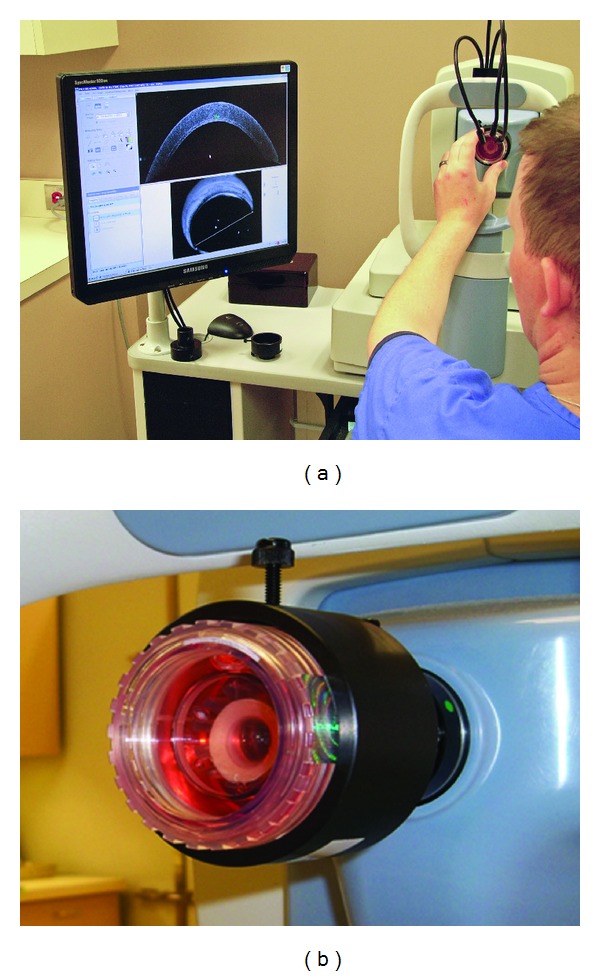
(a) Setup for FD-OCT imaging. The viewing chamber containing the corneoscleral disc is placed in the holding device while the cornea is scanned. In order to minimize the risk of contamination, the cornea remains in medium throughout the scan. (b) View chamber closeup.

After the corneas were identified as having potential anterior stromal pathology by slit lamp examination, each was scanned with the RTVue FD-OCT instrument (Optovue Inc, Fremont, CA, USA) fitted with a cornea adaptor module (CAM) in order to image the cornea. The RTVue-CAM scan rate was 26,000 axial scans per second with 5 *μ*m axial resolution. Each corneoscleral disk was scanned through the transparent window of the corneal viewing chamber which was held in place by a custom-built attachment (Figures 1(a) and 1(b)). A 10 mm line scan was rotated until the opacity could be visualized. Then the depth of the stromal opacity was manually measured using the computer calipers in the RTVue software suite. 

## 3. Results

Seven corneas suspected of having anterior stromal pathology after slit lamp examination were evaluated with the Optovue RTVue FD-OCT. In all seven cases, the Eye Bank Technicians reported that the location and depth of corneal opacity were more sharply defined by OCT than by slit lamp. The measured depth of opacity ranged from 82 *μ*m to 624 *μ*m with an average opacity depth of 291 *μ*m (Table 1). 

**Figure 2:**
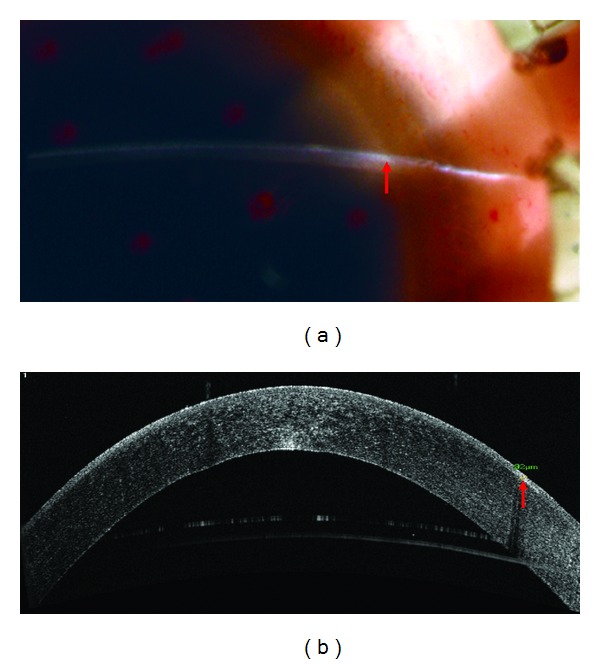
(a) What appears to be anterior stromal pathology as visualized by slit lamp microscopy. (b) FD-OCT evaluation reveals that scarring is limited to the corneal epithelium. The arrow indicates the location of opacities.

**Figure 3:**
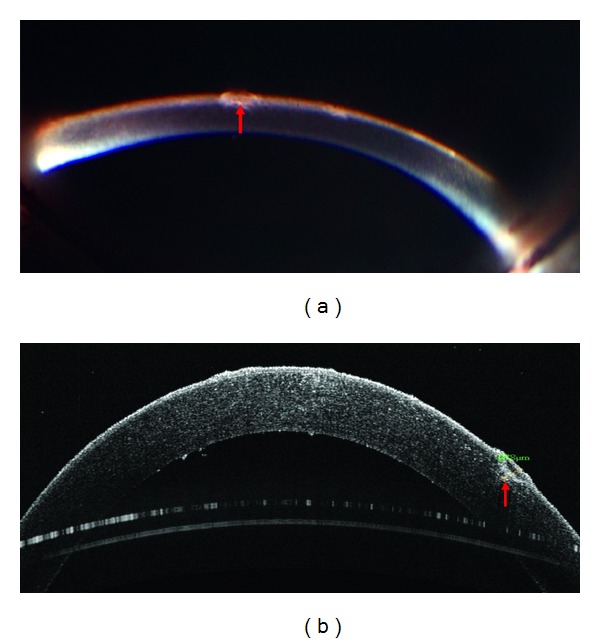
(a) Slit lamp examination suggests that the opacity is limited to the epithelium. (b) OCT examination reveals the presence of significant sub-Bowman's scarring measured to a depth of 278 *μ*m. The arrow indicates the location of opacities.

In two of the seven corneas, the OCT findings were different from the initial slit lamp impressions. In Cornea One, the slit lamp examination gave the impression of an anterior stromal pathology (Figure 2(a)). However, upon OCT evaluation it was shown that the cornea contained no stromal scarring and, with a depth of 82 *μ*m, the opacity was limited to the epithelium (Figure 2(b)). The tissue was eventually used for EK. In Cornea Two, the initial slitlamp examination suggested the presence of an opacity limited to the epithelium of the cornea (Figure 3(a)). It was only after OCT evaluation that significant sub-Bowman's scarring was identified and measured to a depth of 278 *μ*m (Figure 3(b)). This tissue was also designated acceptable for EK but was disposed of after the graft cut failed (Table 1).

**Figure 4:**
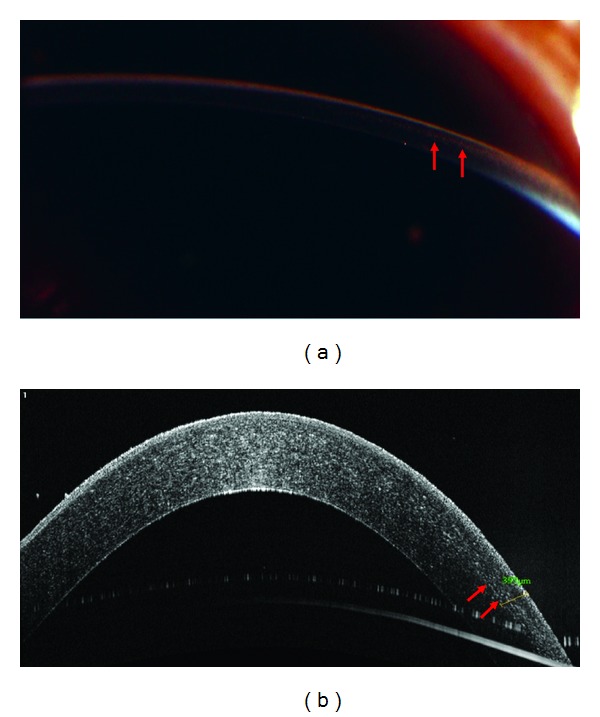
(a) Midstromal Opacity as seen by slit lamp. (b) Midstromal Opacity measured to a depth of 397 *μ*m. The arrow indicates the location of opacities.

OCT imaging of the remaining five corneas confirmed the initial tissue suitability judgments made following slitlamp examination. In these cases, the OCT enabled the technician to make specific depth measurements of stromal opacity. Cornea Three was found to have Radial Keratotomy (RK) scars reaching 624 *μ*m into the stroma. In this case, the cornea was declined for use in transplant because of the donor's medical history. However, with a central corneal thickness of 704 *μ*m (Table 1), the depth of its RK scars would have also prevented it from being used in surgery. Cornea Four contained stromal scarring measured to a depth of 397 *μ*m and was used for EK transplant (Figures 4(a) and 4(b)). Corneas Five, Six, and Seven were each identified as having anterior stromal opacities measuring 224 *μ*m, 212 *μ*m, and 221 *μ*m by OCT, respectively and theses OCT readings agreed with slitlamp examinations. 

## 4. Discussion

Recent years have seen a marked increase in the use of lamellar surgery techniques that selectively replace damaged corneal layers while preserving healthy tissue [20–27]. This increase, and the corresponding decision by the Medical Advisory Board of the EBAA to allow the use of corneas with anterior pathology in EK surgery [1], emphasizes the importance of accurately evaluating each donor cornea. Although the tissue assessment methods currently employed by eye banks are largely successful at detecting the presence of stromal pathology, they provide limited information regarding the location and depth of specific corneal opacities. The capability of FD-OCT to accurately gauge the depth of a corneal opacity has the potential to aid eye banks in efficiently utilizing all available tissue. This study demonstrated that not only does FD-OCT allow Eye Bank Technicians to measure the depth of corneal scarring, but also, when used in conjunction with standard slit lamp microscopy, it may occasionally impact tissue suitability decisions. 

It should be emphasized that we are not suggesting that the information provided by FD-OCT is in itself sufficient for making tissue use judgments. Slit lamp microscopy is still the standard because it allows for a comprehensive survey of the cornea with a multitude of illumination techniques and magnification capabilities, making it the ideal tool for the initial detection and localization of corneal opacities. Rather, the results of this study suggest that FD-OCT is a useful complement to slit lamp examination, a tool for either confirming or discounting ambiguous slit lamp findings. Although the sample size contained in our study is perhaps evidence that ambiguity in slit lamp exams is not a terribly common problem, it is an issue that eye banks need to confront in order to ensure that no donor cornea is needlessly wasted or misused. 

One limitation of the study is the small sample size. Furthermore, the coverage of the OCT line scan is limited. The alignment of the line scan depends on the operator's observation of the area of interest. A better scan pattern would be a 3D wide-angle scan that can map the entire cornea. There is also a small refractive index mismatch between the preservation medium and cornea. The current OCT software does not account for this in its dewarping algorithm which can lead to small errors in corneal thickness measurements.

Moving forward, the correlation between slit lamp findings and FD-OCT imagery may be fertile ground for further investigation. FD-OCT is not currently used to characterize known infections or identify active inflammatory processes. By using FD-OCT to image known contraindications such as active infections, it may be possible to train future Eye Bank Technicians to utilize FD-OCT technology in new and innovative ways. 

Although there is currently no standard procedure for the role of FD-OCT in eye banks, our results demonstrate that its use as an adjunct to standard slit lamp microscopy provides valuable information about stromal pathology that could affect tissue use decisions both now and in the future. 

## Figures and Tables

**Table 1:** Comparison of slit lamp and OCT findings in evaluating donor tissue for transplant suitability and depth of opacity as measured with FD-OCT.

Tissue number	CCT (*µ*m)	Slit lamp finding	FD-OCT finding	Opacity depth (*µ*m)	OCT impact tissue suitability?	Tissue use
1	669	Anterior Stromal Opacity	Epithelial	82	Yes	Endothelial keratoplasty
2	623	Epithelial	Anterior Stromal Opacity	278	Yes	Intended for transplant but microkeratome cut failed—tissue disposed of
3	705	Radial Keratotomy	Radial Keratotomy	624	No	Declined due to medical history
4	641	Midstromal Opacity	Midstromal Opacity	397	No	Endothelial keratoplasty
5	623	Anterior Stromal Opacity	Anterior Stromal Opacity	224	No	Endothelial keratoplasty
6	577	Anterior Stromal Opacity	Anterior Stromal Opacity	212	No	Poor endothelium—tissue disposed of
7	595	Anterior Stromal Opacity	Anterior Stromal Opacity	221	No	Tectonic grafting

CCT: central corneal thickness; FD-OCT: Fourier-domain optical coherence tomography.
